# Atypical presentation of Felty syndrome: Successful management with rituximab therapy—A case report and review of literature

**DOI:** 10.1002/ccr3.9339

**Published:** 2024-08-18

**Authors:** Rabia Iqbal, Ana Colon‐Ramos, Zhenisa Hysenaj, Samridhi Sinha, Shahzaib Nabi, Sabrin Marowa

**Affiliations:** ^1^ Department of Internal Medicine The Brooklyn Hospital Center Brooklyn New York USA; ^2^ Department of Hematology and Oncology The Brooklyn Hospital Center Brooklyn New York USA; ^3^ Zainul Haque Sikder Women's Medical College and Hospital Dhaka Bangladesh

**Keywords:** autoimmune neutropenia, Felty syndrome, hematology, rheumatology, rituximab

## Abstract

The atypical presentation of Felty syndrome, even without arthritis symptoms, needs further evaluation. Timely diagnosis of neutropenia and splenomegaly in patients with rheumatoid arthritis without joint symptoms is crucial for a better prognosis. Despite the rarity of the condition, clinicians should have a high index of suspicion, and multidisciplinary collaboration between rheumatology, hematology, and other specialists is required for accurate diagnosis.

## 1 INTRODUCTION

Felty syndrome (FS), also known as Chauffard‐Still‐Felty syndrome, was first described by Augustus Felty. It is a rare condition diagnosed in patients with long‐standing seropositive rheumatoid arthritis (RA) characterized by an enlarged spleen and neutropenia.^1^ Neutropenia, defined as absolute neutrophil count (ANC) <1500/mm, is FS's hallmark feature. In RA, the patient usually presents initially with arthritis, and the FS commonly develops after many years. However, in highly uncommon instances, FS can manifest before or without any signs of arthritis. Only a few case reports have been published highlighting this unusual presentation of the FS (Table 2). We present a similar case of a young female who initially presented with FS without arthritis. This case report emphasizes the clinical and diagnostic approach to rule out alternate causes of neutropenia with splenomegaly and timely recognition of the atypical presentation of FS.

## 2 CASE REPORT

### 2.1 Case history and presentation

A 24‐year‐old female with a history of migraines and menorrhagia presented to an urgent care center in late summer 2020, complaining of fever, fatigue, and unintentional weight loss. The review of systems was positive for generalized weakness and dizziness. She denied bone pain, chronic diarrhea, recurrent infections, Raynaud's, pleurisy, palpitations, skin rashes, photosensitivity, alopecia, seizures, sicca, or palpable adenopathy. Her social history was remarkable for drinking alcohol every other day from October 2019 to the end of the summer of 2020, cocaine use in the past, and being a former smoker. Her past surgical history is remarkable for bilateral adenoidectomy. Her vital signs were typical. The physical exam was grossly unremarkable.

### 2.2 Methods (differential diagnosis, investigations, and treatment)

A complete blood count (CBC) showed leukopenia at the expense of severe neutropenia, mild normocytic anemia, and normal platelets.

Table 1 shows the initial lab results for the patient.

**TABLE 1:** Initial lab results for the patient.

Laboratory tests	Results	Normal range
WBC	1.4	3.8–10.8 thousand/uL
Hgb	11	11.7–15.5 g/dL
MCV	85.5	80–100 fL
Platelet count	200	140–400
ANC	333	1500–7800 cells/uL
TSH	1.32	0.35–4.94 MICU/mL
Cr	0.6	0.8–1.1 mg/
eGFR	126	>60 mL/min/1.73
Total protein	8.6	6.4–8.3 g/dL
ALT	15	6–55 U/L
AST	17	8–34 U/L
Hep C	Nonreactive	Nonreactive
HIV Ab/Ab combo	Nonreactive	Nonreactive
Zn	0.74	0.66–1.10 mcg/mL
Cu	1.27	0.75–1.45 mcg/mL
B12	513	213–816 pg/mL
Folic acid	10	>5.4 ng/mL
Rheumatoid factor	Positive	Negative
ANA	1:320 (positive)	Negative
CCP	340	<20

Differential diagnosis included infections, sarcoidosis, systemic lupus erythematosus, and myeloproliferative syndromes. Further workup was carried out to determine the cause of decreased neutrophil count. A chemistry panel showed paraproteinemia, normal kidney function, and normal liver enzymes. The infectious panel showed a negative rapid COVID test, HIV negative, hepatitis C antibody test nonreactive, and serologic testing for hepatitis B suggested passive immunity. She was found to have a positive antinuclear antibody (ANA) test and a positive rheumatoid factor (RF). The anti‐cyclic citrullinated peptide (anti‐CCP) was high >340. A peripheral flow cytometry was unremarkable. The patient had a bone marrow biopsy, and aspiration showed a norm cellular marrow with maturing trilineage hematopoiesis, a preserved marrow reserve pool on the two occasions. In addition, there was no morphologic evidence of overt or advanced myelodysplasia, acute leukemia, metastatic neoplasm, plasma cell dyscrasia, or lymphoma.

**FIGURE 1:**
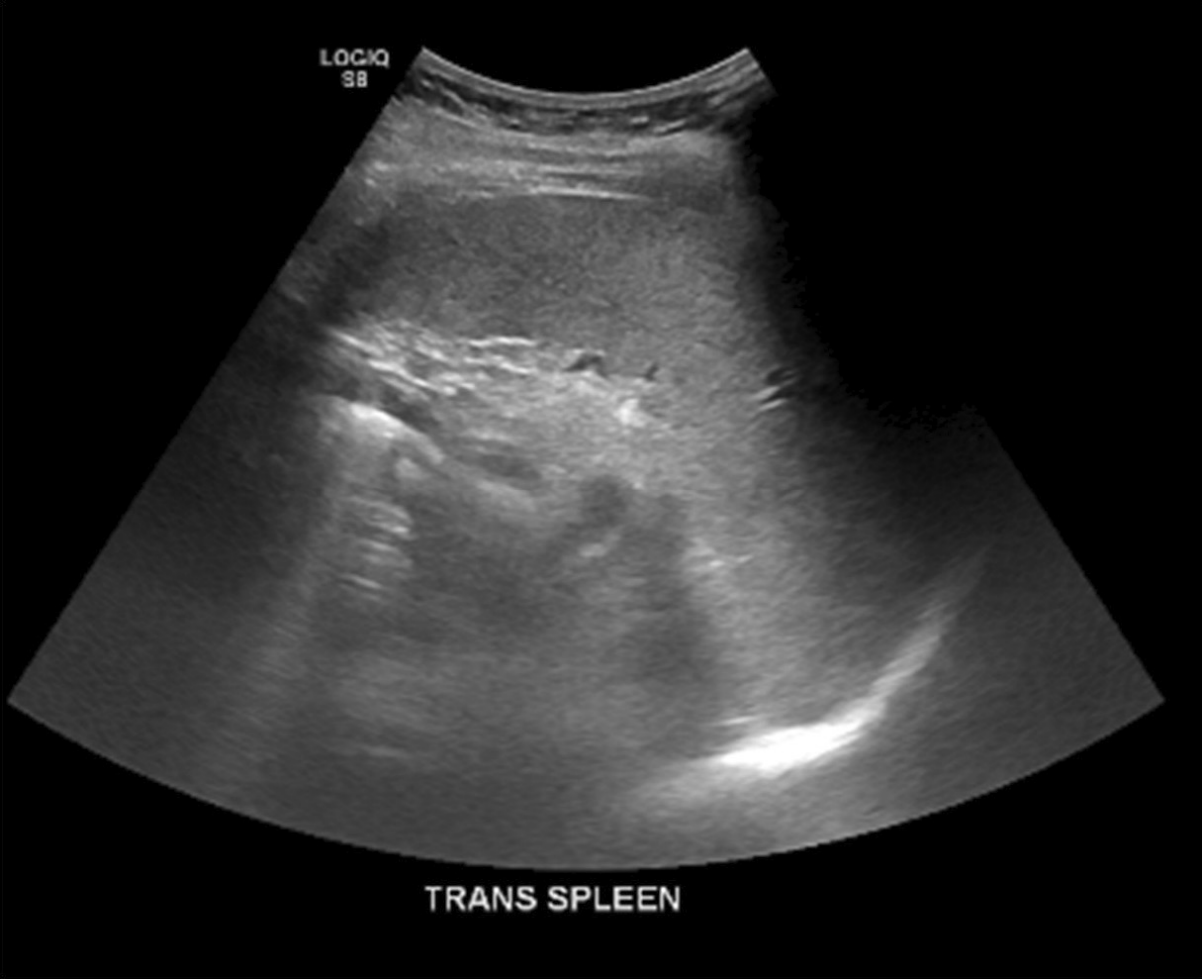
Ultrasound of the abdomen revealing a mildly enlarged spleen. This ultrasound image shows the spleen's dimensions, indicating mild splenomegaly, which is a common finding in patients with Felty Syndrome.

An ultrasound of the abdomen showed a slightly enlarged liver due to possible fatty infiltration, and the spleen measured 11.8 × 4.5 × 13.6 cm, which was mildly enlarged (Figure 1).

Due to the positive RF factor, she was evaluated by rheumatology due to features of FS and diagnosed with rheumatoid arthritis (RA). She initially started prednisone 10 mg daily and later transitioned to hydroxychloroquine 200 mg qd. Despite treatment, the patient reported only mild improvement in her body aches, but her neutropenia and severe fatigue persisted.

Due to her dropping neutrophil count and peak COVID infection, a joint decision to treat her was made with a trial of prednisone 1 mg/kg daily for 2 weeks, resulting in the normalization of the ANC. This response was sustained for 4 months before relapsing. After stopping steroids, the ANC level again dropped. Rituximab was then considered as an alternative for long‐term management of autoimmune neutropenia in the setting of FS.

**FIGURE 2:**
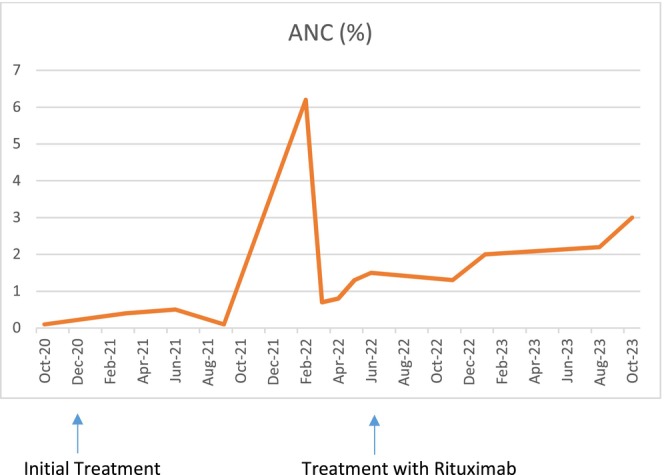
Patient's ANC response over time, first to the initial steroid treatment and subsequently showing significant improvement with rituximab therapy.

**FIGURE 3:**
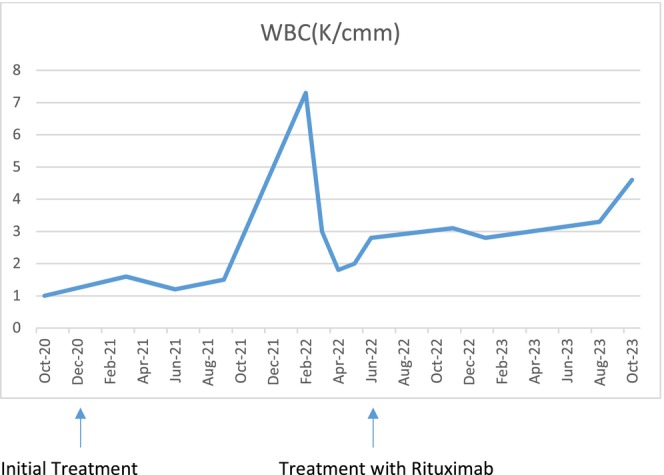
Patient's WBC count changes over time, initially following steroid treatment and subsequently showing marked improvement with rituximab therapy.

Figures 2 and 3 show the trend of ANC and white blood cell (WBC) count after treatment with steroids and rituximab.

### 2.3 Results (outcome and follow‐up)

After the treatment started, her neutrophil count improved. On her follow‐up appointments, her CBC and WBC remained normal, and she reported improvement in her fatigue symptoms. She has not had any hospital admissions or life‐threatening infections, and nonspecific symptoms improved considerably.

## 3 DISCUSSION

FS is an extracellular articular manifestation of RA. The lifetime risk of developing FS for a patient with RA has been estimated to be approximately 1%–3%.^1^ It is primarily diagnosed through clinical evaluation based on the presence of persistent neutropenia and enlarged spleen in chronic RA. It is crucial to emphasize this criterion because diagnosing the condition can be challenging when arthritis symptoms are absent.^4^, ^7^ Arthritis usually occurs for almost 10 or more years before neutropenia is diagnosed.^5^ Beyond this triad, clinical presentations of FS can involve anemia, low platelet counts (thrombocytopenia), recurrent bacterial infections, skin ulcers, unexplained portal hypertension, and an elevated risk for the development of blood‐related cancers like non‐Hodgkin's and Hodgkin's lymphoma.^3^ Though uncommon, documented cases in the literature highlight instances of FS occurring without concurrent articular RA (Table 2). We have presented such a case and briefly reviewed the existing literature.

**TABLE 2:** Literature review of case reports showing atypical presentation of Felty syndrome and its management.

No.	Author	Age	Gender	Splenomegaly/neutropenia	Arthritis/arthralgia at presentation	Treatment	Development of arthritis
1	Chavalitdhamrong D^2^	31	Male	+/+	None	Methotrexate/G‐CSF	N/A
2	Saeed H^4^	55	Female	+/+	None	Filgrastim/methotrexate	Yes
3	Jakez‐Ocampo J^5^	33	Male	+/+	Arthralgia	Prednisone	N/A
4	Jain T^6^	73	Female	+/+	None	Prednisone	N/A
5	Serrano Santiago VE	56	Female	+/+	None	G‐CSF	N/A
6	Aslam F^17^	46	Female	+/+	Arthralgia	Methotrexate	Yes
7	Cornwell GG^14^	56	Male	+/+	None	Splenectomy/cyclophosphamide	None
8	Bradely^15^	66	Female	+/+	None	Splenectomy	Yes
9	Armstrong Case 1^16^	79	Female	+/+	None	N/A	Yes
10	Armstrong Case 2^16^	56	Female	+/+	Yes	N/A	Yes
11	Armstrong Case 3^16^	72	Female	+/+	Yes	N/A	Yes
12	Cycowitz^18^	34	Female	+/+	None	Prednisone	No
13	Muroi^19^	52	Female	+/+	Yes	Splenectomy	N/A
14	Rozin^20^	57	Male	+/+	None	Prednisone, methotrexate	N/A
15	Lagrutta^21^	56	Female	+/+	None	Splenectomy, prednisone, azathioprine	Yes
16	Our case	24	Female	+/+	None	Prednisone/rituximab	No

Table 2 shows a few cases, including ours, in which FS was diagnosed before or simultaneously with the onset of arthritis symptoms.

FS was found to be more common in females. All the patients presented with neutropenia and splenomegaly, and some of them had arthritis initially as well, as shown in the table.

Pathophysiology of neutropenia in FS involves both cellular immunity and humoral immunity. It includes the development of autoantibodies against granulocyte colony‐stimulating factor (G‐CSF) and polymorphonuclear neutrophils (PMN), resulting in apoptosis of neutrophils and neutropenia.^11^

There are no specific criteria for diagnosing FS. It is a clinical diagnosis of RA with splenomegaly and neutropenia. In patients who present without arthritis, positive RF and anti‐CCP antibodies can point toward the possibility of RA, like in our patient. High‐titer RF and anti‐CCP have a specificity of 99.5% for RA.^13^ When the patient tested positive for both factors, she was referred to rheumatology and diagnosed with RA.

FS does not have a specific curative treatment. Literature needs guidelines in the management of FS, although a few articles have highlighted the different strategies. Treatment primarily focuses on improving neutropenia with the treatment of RA, which is the underlying cause.^7^ Methotrexate and rituximab are the preferred DMARDs in patients with FS.^9^ Due to the patient's intention to become pregnant and lack of use of oral contraceptives, methotrexate was not prescribed. Methotrexate has been associated with an increased risk of miscarriage and congenital disabilities, particularly when taken during the first trimester of pregnancy.^22^ Numerous case reports have demonstrated that rituximab can induce a sustained and complete response in autoimmune cytopenias, particularly in cases of FS.^6^, ^8^, ^12^ In our case, the patient responded well to the rituximab treatment. Glucocorticoids can temporarily increase neutrophils as they cause the release of non‐segmented neutrophils into the circulation.^10^ The role of splenectomy is small, although it has been used in refractory cases.

Our patient did not have arthritis; instead, she presented with remarkable fatigue, severe neutropenia in labs, and mild splenomegaly on imaging. Further workup showed high‐titer rheumatoid factor and positive anti‐CCP antibody. The neutropenia normalized with the use of a high dose of steroids but later relapsed. She was then started on rituximab therapy, which improved her neutrophils. However, the patient needs to be closely monitored for neutrophils and the development of arthritis.

While our case report and brief literature review provide valuable insights into FS's atypical presentation and management, it is essential to acknowledge that these findings may only sometimes apply. This limits our report, as it may not apply to all the cases due to individual differences in presentation and response to treatment.

FS is a rare presentation of RA, but persistent neutropenia can lead to life‐threatening infections. Therefore, it is necessary to check for RA and early refer to rheumatology even in the absence of arthritis symptoms. Early detection of FS is crucial for further management and prevention of life‐threatening infections.

## 4 CONCLUSIONS

In conclusion, this case highlights the present atypical manifestation of FS, underscoring the challenges in managing this rare condition. The case findings emphasize the importance of considering diverse clinical manifestations of FS. We also emphasize the importance of ruling out other causes of neutropenia and splenomegaly. If left untreated, neutropenia can lead to the development of life‐threatening infections. Awareness among healthcare professionals, particularly hematologists, is crucial for timely recognition and appropriate intervention, ensuring optimal care for patients with this complex autoimmune condition.

## AUTHOR CONTRIBUTIONS

**Rabia Iqbal:** Conceptualization; data curation; formal analysis; writing – original draft; writing – review and editing. **Ana Colon‐Ramos:** Conceptualization; data curation; resources; supervision; writing – review and editing. **Zhenisa Hysenaj:** Conceptualization; resources; supervision; writing – review and editing. **Samridhi Sinha:** Conceptualization; supervision; writing – review and editing. **Shahzaib Nabi:** Conceptualization; data curation; resources; supervision; writing – review and editing. **Sabrin Marowa:** Conceptualization; data curation.

## FUNDING INFORMATION

No funding was received for our case presentation.

## CONFLICT OF INTEREST STATEMENT

The authors declare no conflicts of interest.

## CONSENT

Written informed consent was obtained from the patient to publish this report in accordance with the journal's patient consent policy.

## Data Availability

This study involves a review of existing case reports from published literature. All data referenced and analyzed in this manuscript are publicly available from the respective journal articles and databases cited within the text. Data sharing is not applicable to this article as no new data were created or analyzed in this study.
